# Isolation and Characterization of Microsatellite Loci in the Chinese Cobra *Naja atra* (Elapidae)

**DOI:** 10.3390/ijms12074435

**Published:** 2011-07-07

**Authors:** Long-Hui Lin, Lu-Xi Mao, Xia Luo, Yan-Fu Qu, Xiang Ji

**Affiliations:** 1 Hangzhou Key Laboratory for Animal Adaptation and Evolution, School of Life Sciences, Hangzhou Normal University, Hangzhou 310036, Zhejiang, China; E-Mail: linlh@yahoo.cn; 2 Jiangsu Key Laboratory for Biodiversity and Biotechnology, College of Life Sciences, Nanjing Normal University, Nanjing 210046, Jiangsu, China; E-Mails: maoluxi@hotmail.com (L.-X.M.); imwx-q@163.com (Y.-F.Q.); 3 School of Life Science, Anhui University, Hefei 230039, Anhui, China; E-Mail: lxsm1986@163.com

**Keywords:** *Naja atra*, Elapidae, microsatellites, PCR primers, polymorphism

## Abstract

We characterize thirteen polymorphic microsatellite loci isolated from *Naja atra* genomic libraries, which were enriched for AC-motif microsatellites. The thirteen loci were screened on a group of 48 individuals from two populations, one in Yong’an and the other in Ganzhou. These markers revealed a relatively high degree of genetic diversity (4–12 alleles per locus) and heterozygosity (Ho ranged from 0.213–0.854 and He ranged from 0.301–0.838). Tests for departure from Hardy-Weinberg equilibrium and for linkage disequilibrium were conducted for each of the two populations separately. After sequential Bonferroni correction, none of the 13 loci showed significant departures from Hardy-Weinberg equilibrium. Hierarchical analysis of molecular variance indicated that a small but significant (*P* < 0.001) proportion (16.0%) of the total variation in the microsatellite DNA data were attributable to differences among populations, indicating geographical structuring and restricted gene flow. It could be attributable to the Wuyi mountains in the area having a sufficiently isolating effect to significantly reduce gene flow. Our microsatellite data also showed a low *N*_m_ (1.31) value in the two populations from mainland China. Thus, the Yong’an and Ganzhou populations could be treated as distinct evolutionarily significant units (ESUs). The high level of polymorphism revealed by these microsatellite markers will be useful for the study of gene flow, population structure and evolutionary history of *N. atra.*

## 1. Introduction

**Figure 1:**
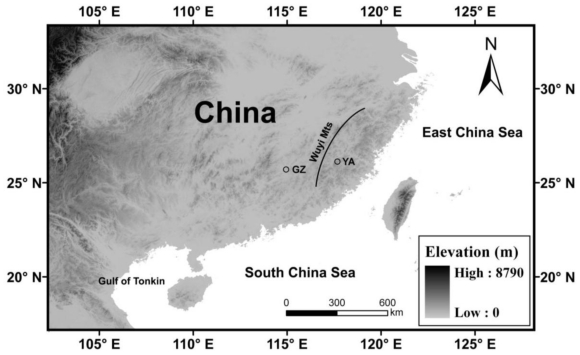
Map of Southeast China, showing the two localities where the cobras used in this study were collected. GZ: Ganzhou; YA: Yong’an.

The Chinese cobra (*Naja atra*) is an oviparous elapid snake with a distribution covering southeastern China (including Taiwan, Hongkong and Hainan) and northern Vietnam (see Figure 1) [1]. *Naja atra* was one of the snakes most commonly found in China thirty years ago. Nowadays, it is regarded as a highly vulnerable species according to the China Red Data Book of Endangered Animals [2], largely because cobras have been over-hunted by local people for their meat and skin, which are used in medicine and handwork. There are realistic threats of local extinction in some southern provinces such as Guangdong and Hainan [2]. To develop effective conservation strategies for the cobra, we need a better understanding of gene flow, population structure and evolutionary history of this species. One approach for achieving this goal is to use specific molecular markers. Among them, microsatellites or short, tandemly repeated base-pair sequences are the most useful due to the high variability caused by changes in their repeat number [3]. Here, we describe the isolation and characterization of thirteen microsatellite markers in *N. atra*.

## 2. Material and Methods

Forty-eight individuals (*n* = 24 for each population) used in this study were collected from two populations, one in Yong’an (Fujian Province) and the other in Ganzhou (Jiangxi Province). Fresh tissue (tail muscle) samples were used to extract total genomic DNA using EasyPure Genomic DNA Extraction Kit (TransGen Biotech). DNA was resuspended in TE buffer (10 mM Tris-HCl, pH 8.0, 0.1 mM EDTA). Genomic DNA was used to construct a library enriched for AC-motif microsatellite sequences. The enrichment protocol essentially followed the FIASCO (Fast Isolation by AFLP of sequences COtaning repeats) [4] with minor modifications. Genomic DNA was digested with *Mse* I and ligated to *Mse* I AFLP adaptor. The digestion-ligation mixture was diluted (1:10), and directly amplified in a total volume of 20 μL with primer *Mse* I-N. The product from this step was mixed 1 μL (about 20 pmol) of a 5′-biotinylated (AC)_10_ oligonucleotide probe in a total volume of 40 μL (0.1 × saline sodium citrate buffer) at 68 °C for 1 h. The DNA fragment (AC)_10_ complex were obtained by adsorption of streptavidin and separated by a magnetic field from the hybridization buffer. The complex fragments were ligated into PMD19-T vector and transformed into competent cells (DH5α). Cells were plated on “blue-white” of Luria-Bertani plates. In total, 384 positive colonies were selected by polymerase chain reaction using nonbiotinylated (AC)_10_ probe primer and universal M13 primers. Those that produced a two-band pattern in agarose electrophoresis were considered to contain a microsatellite repeat.

For the microsatellites sequences containing adequate flanking regions, PCR primers were designed using CID software [5]. From these primers, 28 produced consistent amplification of loci in eight tested individuals from Yong’an population. All PCRs were performed with the following conditions (in 20-μL volume): 100–200 ng of genomic DNA, 10 μL 2 × EasyTaq PCR SuperMix polymerase (TransGen Biotech), 0.5 μM of each primer; 5 min denaturation at 95 °C; 32 cycles of 30 s at 95 °C, 1 min at specific annealing temperatures, and 1 min at 72 °C (Table 1); and a final extension of 72 °C for 10 min. Amplification and polymorphism were assessed in 48 individuals of *N. atra* from the Yong’an and Ganzhou populations. All PCR products were genotyped using an ABI PRISM 3730 genetic analyzer (Applied Biosystems) along with Rox GS350 size standard, and analyzed with GENEMARKER (version 1.85, Applied Biosystems).

The number of alleles at each polymorphic locus, their size range and observed and expected heterozygosities were calculated using CERVUS 3.0 software [6]. Deviation from Hardy-Weinberg equilibrium (HWE) and linkage disequilibrium (LD) at each locus was calculated using GENEPOP 4.0.10 [7,8]. Hierarchical analysis of molecular variance (AMOVA) with 1,000 permutations was performed to examine partitioning of genetic diversity within and among populations using ARLEQUIN 3.11 [9].

## 3. Results and Discussion

Details of the microsatellite loci and variability measures across 48 individuals of the Chinese cobra are summarized in Table 1. The 13 sequences containing a microsatellite locus were deposited in GenBank (HQ881484-HQ881496). The number of alleles ranged from 4 to 12; observed heterozygosity (Ho) ranged from 0.213–0.854, and expected heterozygosity (He) ranged from 0.301–0.838 (Table 1).

Tests for departure from HWE and for LD were conducted for each of the two populations separately. After sequential Bonferroni correction [10], none of the 13 loci showed significant departures from Hardy-Weinberg equilibrium for each of the two populations. Two (from the Yong’an population) and one (from the Ganzhou population) out of 78 pairwise comparisons exhibited significant linkage disequilibrium respectively following sequential Bonferroni correction.

AMOVA indicated that a small but significant (*P* < 0.001) proportion (16.0%) of the total variation in the microsatellite DNA data were attributable to differences among populations, indicating geographical structuring and restricted gene flow. It could be attributable to the Wuyi mountains in the area having a sufficiently isolating effect to significantly reduce gene flow (see Figure 1). A significant (*P* < 0.001) proportion of variation also occurred within populations (84.0%). Previous work based on data with the mitochondrial control region sequences of the Chinese cobra in Taiwan showed low *N*_m_ values (0.31–2.00) between populations, indicating low levels of gene flow [11]. Accordingly, the four populations in Taiwan were treated as distinct evolutionarily significant units (ESUs) [11]. Our microsatellite data also showed a low *N*_m_ (1.31) value between the two populations from mainland China. The results of AMOVA and the low *N*_m_ value between the two populations indicated that the Yong’an and Ganzhou populations should be treated as distinct ESUs. Given the existence of distinct ESUs, reintroducing confiscated cobras from the illegal trade back into the wild should be conducted with caution to prevent artificial gene flow.

In conclusion, the microsatellite loci presented in this study will be useful for genetic studies in *N. atra* especially directed toward a finer-scaled understanding of gene flow, population structure and dispersal dynamics within and between population groups.

## Figures and Tables

**Table 1:** Characteristics of 13 *Naja atra* microsatellites DNA loci: locus designation, repeat motif, primer sequences, allele size range (bp), annealing temperature, *T_a_* (°C), observed number of alleles (*N_a_*) and effective number of alleles (*N_e_*), average observed (*H_o_*) and expected heterozygosity (*H_e_*), Polymorphic information content (*PIC*), number of individuals successfully genotyped (*N*), and GenBank Accession number.

Locus	Repeat motif	Primer sequences (5′-3′)	Size range (bp)	*T_a_* (°C)	*N_a_*	*N_e_*	*H_o_*	*H_e_*	*PIC*	*N*	Accession No.
p26	(TGTA)_11_	F:5′TAMRA-TGCGAAATAACCCATTCTAATC R:5′-TCTAGATGCTTATATGACCACAGG	200–242	55	9	4.378	0.525	0.781	0.743	40	HQ881486
p92	(AC)_14_	F:5′HEX-AACCTCTCCCTGAATCAACTAG R:5′-GCAGATATAGCACACTTGTTTG	145–171	55	7	2.344	0.512	0.580	0.492	43	HQ881492
p122	(AC)_16_	F:5′FAM-TGAATGAAGGAACAGCACAATC R:5′-AACAGGAGCGAAGAGGTTTC	140–170	55	4	2.090	0.500	0.527	0.461	44	HQ881490
p121	(GT)_18_	F:5′TAMRA-AGTCAACTGAATCTTCTCCATC R:5′-CAACTCCACTTCCCCAGAC	173–208	55	6	2.620	0.213	0.625	0.590	47	HQ881493
p8	(GT)_15_	F:5′FAM-TTCATTGAGTGGTGAGTGAGC	118–146	55	8	2.012	0.362	0.508	0.460	47	HQ881488
		R:5′-CTTCAGACCCTTCTCTTTATTACTC									
p124	(CA)_19_	F:5′TAMRA-CACTGTTGGTCTGAAAATCTCTAT R:5′-GTTTTCAGTTTGTTTGTCAATGTC	103–140	55	10	5.863	0.417	0.838	0.807	48	HQ881487
p88	(AC)_14_	F:5′HEX-CACACAAACAATCGCTCTACAC R:5′-AGCCTTCCAGACATCAAATCAG	50–77	55	6	1.936	0.396	0.489	0.454	48	HQ881484
p87	(GT)_22_	F:5′FAM-AAATCCCAGTAGAGGTTTGTTC R:5′-GGACAGTGGTCGTCACAC	102–144	55	8	4.267	0.417	0.774	0.732	48	HQ881489
p265	(TG)_19_	F:5′HEX-GCACACAGGGTCTTCATTATTG R:5′-TCCACAGCGAAACTCATCAG	128–164	55	10	5.260	0.646	0.818	0.785	48	HQ881491
p132	(AC)_20_	F:5′FAM-TTCACCAGGTTCCAATGTTCC R:5′-AGTCAGCCATTGTTCACTCTG	83–121	56	5	1.425	0.319	0.301	0.280	47	HQ881485
p22	(CA)_22_	F:5′TAMRA-CAAGGGGACAGTGGTCTTC R:5′-TCTGGGCAAATGATGAAAATCC	52–94	55	6	2.628	0.468	0.626	0.563	47	HQ881494
p262	(AC)_16_	F:5′HEX-CACTGGAGCTGGACACTTG R:5′-TCATTTAGAGCAAAGGTGATGC	107–137	55	12	5.804	0.854	0.836	0.810	48	HQ881495
p140	(TG)_16_	F:5′FAM-GGAAAGATGGAAATGGCTTCAC R:5′-ATTCGGTTTGGTGGCAGAC	142–173	55	7	2.122	0.333	0.534	0.493	48	HQ881496
